# Containment of COVID-19 cases among healthcare workers: The role of surveillance, early detection, and outbreak management

**DOI:** 10.1017/ice.2020.219

**Published:** 2020-05-11

**Authors:** Liang En Wee, Xiang Ying Jean Sim, Edwin Philip Conceicao, May Kyawt Aung, Jia Qing Goh, Dennis Wu Ting Yeo, Wee Hoe Gan, Ying Ying Chua, Limin Wijaya, Thuan Tong Tan, Ban Hock Tan, Moi Lin Ling, Indumathi Venkatachalam

**Affiliations:** 1 Singhealth Infectious Diseases Residency, Singapore; 2 Department of Infectious Diseases, Singapore General Hospital, Singapore; 3 Department of Infection Prevention and Epidemiology, Singapore General Hospital, Singapore; 4 Department of Clinical Quality and Performance Management, Singapore General Hospital, Singapore; 5 Department of Occupational and Environmental Medicine, Singapore General Hospital, Singapore

## Abstract

**Objective:**

Staff surveillance is crucial during the containment phase of a pandemic to help reduce potential healthcare-associated transmission and sustain good staff morale. During an outbreak of SARS-COV-2 with community transmission, our institution used an integrated strategy for early detection and containment of COVID-19 cases among healthcare workers (HCWs).

**Methods:**

Our strategy comprised 3 key components: (1) enforcing reporting of HCWs with acute respiratory illness (ARI) to our institution’s staff clinic for monitoring; (2) conducting ongoing syndromic surveillance to obtain early warning of potential clusters of COVID-19; and (3) outbreak investigation and management.

**Results:**

Over a 16-week surveillance period, we detected 14 cases of COVID-19 among HCWs with ARI symptoms. Two of the cases were linked epidemiologically and thus constituted a COVID-19 cluster with intrahospital HCW–HCW transmission; we also detected 1 family cluster and 2 clusters among HCWs who shared accommodation. No transmission to HCWs or patients was detected after containment measures were instituted. Early detection minimized the number of HCWs requiring quarantine, hence preserving continuity of service during an ongoing pandemic.

**Conclusions:**

An integrated surveillance strategy, outbreak management, and encouraging individual responsibility were successful in early detection of clusters of COVID-19 among HCWs. With ongoing local transmission, vigilance must be maintained for intrahospital spread in nonclinical areas where social mingling of HCWs occurs. Because most individuals with COVID-19 have mild symptoms, addressing presenteeism is crucial to minimize potential staff and patient exposure.

In the coronavirus disease 2019 (COVID-19) pandemic, nosocomial transmission has emerged as a significant concern; almost one-third of an initial cohort of COVID-19 patients were healthcare workers (HCWs) and hospitalized inpatients.^1^ Given that hospitals are often the epicenters of newly circulating infections, HCWs are at high risk of acquiring infectious diseases.^2^ Furthermore, HCW contacts can differ substantially from those of individuals in the community; a study of HCWs reported more work-related contacts than community-based working adults.^3^ Thus, HCWs may be at higher risk of acquiring infections due to the nature of their work.^3^ Monitoring infective symptoms among HCWs might be a feasible means of surveillance for emerging infections^2,4^; however, surveillance is time-consuming.^4^ Hospital-based syndromic surveillance can facilitate early detection of a healthcare-associated outbreak,^5^ and a surveillance system for HCWs must be supported by a comprehensive outbreak management strategy^6,7^ to achieve containment.

In Singapore, a globalized Asian city-state, the first imported case of COVID-19 was reported on January 23, 2020; followed by the first documented case of local transmission on February 4, 2020.^8^ Previously, during an outbreak of severe acute respiratory syndrome (SARS) in Singapore a decade earlier, staff surveillance, such as mandatory temperature monitoring, was part of our healthcare system’s containment strategy.^4,9^ However, at the onset of the COVID-19 epidemic, we recognized several key differences. First, unlike SARS, in which fever predominated,^10^ fever may not occur in all patients with COVID-19 on initial presentation.^11^ Thus, syndromic surveillance had to be expanded beyond temperature monitoring to include respiratory symptoms as well. Additionally, in SARS, relatively few individuals were asymptomatic and most required hospitalization,^10^ leading to a significant risk of patient–HCW transmission.^12^ However, with SARS-CoV-2, given that a greater proportion of individuals are asymptomatic and well enough to remain in the community,^13^ the possibility that HCWs might bring SARS-CoV-2 into the healthcare system from their community contacts was a cause for concern. Indeed, in the initial phases of the SARS-CoV-2 outbreak in Singapore, community transmission, rather than healthcare-associated transmission, predominated.^14^ Our institution adopted a 3-pronged strategy to contain COVID-19 among our hospital workforce: (1) strengthening centralized reporting of HCWs with acute respiratory illness, (2) conducting surveillance for early warning signs of potential clusters, and (3) performing epidemiology investigation and outbreak management when cases of COVID-19 were detected among HCWs. During an ongoing outbreak of SARS-CoV-2 with community transmission, our institution successfully utilized this strategy to detect and contain a cluster of COVID-19 cases among HCWs.

## Methods

### Institutional setting and study period

Our institution, Singapore General Hospital (SGH) is the largest public tertiary-care hospital in Singapore, with 1,785 beds. SGH has a total of 9,322 HCWs: 1,187 medical staff, 3,914 nursing staff, 1,777 allied health personnel, and 2,444 ancillary personnel. SGH shares a campus with other institutions under the same hospital group: namely the Singapore National Eye Centre (910 HCWs), National Cancer Centre, Singapore (1,092 HCWs), National Heart Centre, Singapore (1,328 HCWs), and National Dental Centre, Singapore (513 HCWs). The staff of these institutions also utilize the staff clinic at SGH and share on-campus facilities. We evaluated our institution’s strategy for early detection and containment of potential COVID-19 outbreaks among HCWs working on the same hospital campus over a 16-week study period (January 1 to April 22, 2020).

### Strengthening ground-level reporting and compliance for HCWs with acute respiratory illness during the SARS-CoV-2 outbreak

Beginning January 1, 2020, in line with guidance from our local ministry of health, our hospital group activated a range of surveillance measures. Staff advisories were disseminated to advise HCWs to immediately report to our institution’s staff clinic during office hours or to the emergency department (ED) after hours, should they have symptoms of acute respiratory illness (ARI). HCWs were also advised not to come to work if they were unwell with ARI symptoms. Supervisors were also encouraged (1) to notify our institution’s department of infection prevention and epidemiology (IPE), should they notice above-average numbers of HCWs in their work area reporting sick due to ARI and (2) to ensure that these HCWs report to the staff clinic for evaluation. All HCWs campus-wide were required to measure their temperature twice daily and record it in an electronic surveillance system, and compliance was monitored.

### Workflow for HCWs reporting sick with ARI during the SARS-CoV-2 outbreak

The HCWs who reported to the staff clinic or ED with ARI symptoms and who did not require admission were given 5 days of medical leave and were instructed to rest at home. If the symptoms did not resolve after 5 days, they were instructed to return to the staff clinic, where oropharyngeal swabs were obtained for SARS-CoV-2 testing and medical leave was subsequently extended by another 5 days. To mitigate potential exposure in the staff clinic, all HCWs attending the staff clinic with ARI symptoms were given surgical masks on arrival and were seen in a separate area. All HCWs seeing ARI patients in the staff clinic used N95 masks, eye protection (eg, goggles and face shield), gown, and gloves when seeing patients and taking respiratory specimens because patients with ARI symptoms are suspected for COVID-19, given ongoing community transmission. Testing for SARS-CoV-2 RNA was conducted using qualitative real-time reverse transcription PCR (RT-PCR) and was performed by our institution’s molecular diagnostics laboratory.

### Staff surveillance during the SARS-CoV-2 outbreak with ongoing local transmission

Temperature surveillance data and information on HCW visits to the staff clinic or ED for ARI symptoms were as collected daily and were collated by our IPE department to analyze trends and perform syndromic surveillance. This information was also aggregated with data from our human resources department to identify potential clusters of staff with fever or ARI symptoms. Heat maps were plotted to cluster HCWs with fever or ARI symptoms, with respect to time of symptom onset and individual working locations.

### Epidemiology investigation and outbreak management

The IPE department conducted epidemiology investigations when the following triggers were activated: (1) when ground-level supervisors reported above-average numbers of staff in their work areas reporting sick for ARI; (2) when heat maps of surveillance data suggested that an unusual aggregation of HCWs in a specific location had fever or ARI symptoms, defined as ≥3 staff from the same reporting location presenting with ARI symptoms to the staff clinic or ED for evaluation or having fever (>38°C) based on temperature surveillance, within 72 hours of each other, based on norms set in previous local studies;^4^ and (3) when COVID-19 was confirmed in an HCW. If ground-level supervisors reported above-average numbers of HCWs with ARI, or if a cluster of HCWs with fever or ARI in a specific location was detected on heat maps, a list of the individual HCWs was generated and they were asked to present to the staff clinic for further evaluation and SARS-CoV-2 testing.

Upon detection of a confirmed case of COVID-19 among HCWs, contact tracing was conducted within 24 hours to identify other HCWs and patients who had come into contact with the confirmed case. Risk stratification was conducted based on the duration of contact, nature of activity, and personal protective equipment (PPE) worn by the HCW at the time of contact. An exposed HCW was defined as having had contact within 2 m of the index case for a cumulative time of ≥15 minutes; unprotected exposure was defined as having used no PPE during exposure.^15^ HCWs and patients deemed to have significant unprotected exposure based on our local ministry of health’s guidelines were placed under a 14-day quarantine (home isolation), during which they were monitored for symptoms (eg, cough, dyspnea, and myalgia) and twice-daily temperature measurements were submitted via our institution’s electronic surveillance system. If exposed patients or HCWs developed symptoms, swabs were sent for SARS-CoV-2 testing. HCWs with contact not amounting to significant unprotected exposure were allowed to continue work but were placed on daily active phone surveillance by our IPE department. If ARI symptoms developed within 14 days from the date of exposure, individuals on phone surveillance were instructed to return to the staff clinic for further evaluation and SARS-CoV-2 testing. Epidemiology investigations that confirmed 2 or more linked cases of COVID-19 among HCWs with suspected intrahospital transmission constituted an outbreak, and specific containment measures were instituted by our hospital disease outbreak taskforce based on the recommendations of our IPE department.

## Ethics approval

This descriptive study was based on data collected by the hospital’s IPE department as part of our outbreak management strategy, and ethics approval was not required under our hospital’s institutional review board guidelines.

## Results

### Centralized reporting for ARI among HCWs during the SARS-CoV-2 outbreak

Over the 16-week study period (January 1, 2020, to April 22, 2020), a total of 2,250 HCWs presented to the staff clinic or emergency department of our institution with a diagnosis of ARI. Among them, 2,090 (92.8%) were examined at the staff clinic. Overall, 1,642 (72.9%) were tested for SARS-CoV-2, and 9 (0.54%) were positive. Of the 14 confirmed COVID-19 cases among HCWs on our hospital campus, 9 (64.3%) were detected at our institution’s staff clinic or ED, due to the emphasis placed on centralized reporting. Over the same period, 400 cases of COVID-19 were managed at our institution, and 10,141 cases were reported nationwide.

### Syndromic surveillance and monitoring for potential clusters among HCWs

**Fig. 1.**
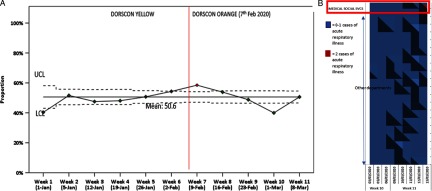
Surveillance for acute respiratory illness among healthcare workers (HCWs) at an acute- and tertiary-care hospital during a COVID-19 epidemic, prior to the detection of a cluster of COVID-19 cases among HCWs. (A) Among HCWs, percentage of staff clinic and emergency department visits attributed to acute respiratory illness over an 11-week period. (B) Heat maps illustrate clustering of HCWs with symptoms of acute respiratory illness, clustered by duration of symptoms and by reporting location (departments), with a focus on the medical social work department over weeks 10 and 11 of the study period, prior to the detection of a staff cluster among medical social workers. The disease outbreak response system condition (DORSCON) is a color-coded framework used by our local ministry of health to indicate the severity of the current outbreak situation and to activate a series of interventions. DORSCON yellow indicates that disease is severe but is occurring outside Singapore, and DORSCON orange indicates that disease is severe, with ongoing local transmission, but it is currently being contained. Note. UCL, upper limit of confidence; LCL, lower limit of confidence.

Of the 4,411 HCW visits to the staff clinic or ED over the study period, 2,250 (51.0%) were for ARI symptoms. We observed a slight increase in the proportion clinic visits for ARI (Fig. 1) after our local ministry of health upgraded the disease outbreak response system condition (DORSCON) level from yellow to orange, signaling the presence of possible ongoing local transmission in Singapore. Over the study period, heat maps of surveillance data flagged 4 potential clusters for investigation (ie, ≥3 staff from the same reporting location presenting with ARI symptoms within 72 hours of each other): 6 nurses in an inpatient ward; 4 staff in the outpatient retail pharmacy; 12 nurses in the ambulatory endoscopy center; and 4 surgical staff. None of the HCWs in these clusters tested positive for SARS-CoV-2.

### Case detection and epidemiology investigation

Over the 16-week study period, 14 HCWs across the hospital campus tested positive for SARS-CoV-2. The demographics of HCWs diagnosed on our hospital campus with COVID-19, as well as the numbers of potentially exposed patients and HCWs, are provided in Table 1. All HCWs confirmed to have COVID-19 were admitted to an isolation ward while epidemiology investigations were conducted. Of these 14 cases, 10 (71.4%) were from local transmission. All presented with upper respiratory tract infection alone, and most were afebrile. Moreover, 10 of the 14 HCWs (71.4%) were nonmedical personnel: 2 medical social workers (MSWs), 1 psychologist, 2 researchers, 1 administrative staff, and 4 cleaners. No other patients exposed to these 14 cases developed COVID-19 despite being followed for 2 weeks. In most of them, patient exposure was limited because they worked in non–patient-facing roles, and throughout the hospital, surgical masks were a mandatory minimum in patient-facing areas.

**Table 1.** Demographics, Epidemiology Investigations and Number of Potentially Exposed Patients and Healthcare Workers (HCWs), Among Confirmed COVID-19 Cases in HCWs at an Acute- and Tertiary-Care Hospital During an Outbreak of SARS-CoV-2 (N=14)

Case No.	Demographics and Occupation	Febrile Prior to Detection of Illness	Imported Case vs. Locally Transmitted Case	Epidemiology Linkage	Attended Staff Clinic or ED	Returned to Work After Symptom Onset/Prior to Symptom Resolution	No. of HCWs Who Came Into Contact With Cases	No. of HCW With Significant Unprotected Contact Requiring Quarantine	No. of Patients Who Came Into Contact With Cases	No. of Patients With Significant Unprotected Contact Requiring Quarantine
1^a^	26 yo woman, medical social worker	Afebrile	Local transmission	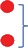 Linked	Yes	Yes (prior to resolution)	86	48	8	0
2^a^	31 yo woman, medical social worker	Afebrile	Local transmission		Yes	No	11	11	2	0
3	32 yo woman, psychologist	Febrile	Imported case	Contact with community COVID-19 case	Yes	Yes (after symptom onset)	4	1	1	1
4^b^	58 yo man, doctor	Afebrile	Imported case	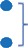 Linked	Yes	No	1	0	4	0
5^b^	55 yo woman, researcher	Afebrile	Imported case		No, directly admitted in view of contact	No	0	0	0	0
6	29 yo woman, researcher	Afebrile	Local transmission	Unlinked	Yes	No	7	2	0	0
7	37 yo woman, nurse	Afebrile	Imported case	Unlinked	Yes	Yes (after symptom onset)	5	2	0	0
8	22 yo woman, nurse	Afebrile	Local transmission	Family cluster; transmission outside of hospital	No, admitted to another institution (with family)	No	0	0	0	0
9	27 yo woman, administrative staff	Afebrile	Local transmission	Unlinked	Yes	Yes(after symptom onset)	7	3	0	0
10	22 yo woman, nurse	Afebrile	Local transmission	Family cluster; transmission outside of hospital	No, admitted to another institution (with family)	No	5	5	3	0
11^c^	28 yo man, cleaner	Febrile	Local transmission	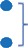 Linked	No, tested at another institution	Yes (after symptom onset)	1	1	0	0
12^c^	27 yo man, cleaner	Febrile	Local transmission		Yes	Yes (after symptom onset)	1	1	0	0
13^d^	27 yo man, cleaner	Afebrile	Local transmission	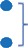 Linked	Yes	Yes (prior to resolution)	1	1	0	0
14^d^	36 yo man, cleaner	Febrile	Local transmission		No, directly admitted in view of contact	Yes (after symptom onset)	1	1	0	0

Note. ED, emergency department.

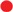
=Linked case, with intra-hospital transmission.

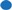
=Linked case, with intra-hospital transmission in a non-clinical area.

a Staff cluster with likely intrahospital transmission in a nonclinical area: shared an office, did not have contact outside of work.

b Staff cluster with transmission outside the hospital. Cases 4 and 5 were part of a family cluster of imported cases (N=3), who tested positive after returning from overseas.

c Staff cluster with transmission outside the hospital. Cases 11 and 12 originally stayed in different rooms of a dormitory that was a known COVID-19 cluster. They were initially asymptomatic, so they were moved into shared temporary accomodation (a room with an attached toilet). Cases 11 and 12 went to work separately and worked in different blocks of the hospital; they had no contact while at work.

d Staff cluster with transmission outside the hospital. Cases 13 and 14 originally stayed in different rooms of a dormitory that was a known COVID-19 cluster. They were initially asymptomatic, so they were moved into shared temporary accomodation (a room with an attached toilet). Cases 13 and 14 went to work separately and worked in different blocks of the hospital; they had no contact while at work.

Among the 14 HCWs with COVID-19, 4 clusters were linked through epidemiology investigations. Of the 4 clusters, most were likely linked via transmission outside the hospital. One cluster was a family cluster (1 doctor and 1 researcher), and 2 clusters among hospital cleaners consisted of HCWs who shared off-site accommodation (single room with en suite bathroom) but worked in different hospital areas and did not mingle while at work. A cluster of 2 MSWs with COVID-19 and suspected intrahospital spread was detected at the end of week 11 on March 13, 2020, as part of epidemiology investigations. The results of syndromic surveillance and heat maps in the period leading up to the detection of the cluster are shown in Figure 1. The 2 MSWs shared the same office and did not interact with each other outside of work; hence, this constituted a COVID-19 cluster with probable HCW-HCW transmission within the hospital in a non-clinical area.

### Outbreak investigation and management

**Fig. 2.**
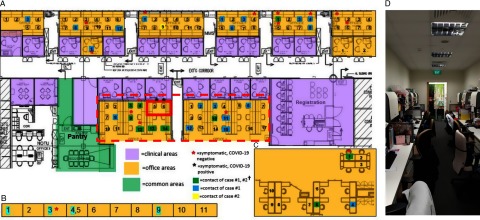
Distribution of healthcare workers (HCWs) with significant contact history, symptomatic HCWs, and office layout, during detection of a cluster of COVID-19 cases among HCWs. (A) Main medical social services office layout. (B) Series of single-room offices used by senior medical social workers located on the same floor. (C) Off-site medical social services office located in another office tower. (D) Typical layout in main medical social services office at the time of the outbreak. ^†^A total of 49 staff were placed on quarantine (home isolation) based on significant unprotected contact with the 2 cases. Of these 49 staff, 10 had significant unprotected contact with both case 1 and case 2; 23 staff had significant unprotected contact with case 1 only; and 1 had significant unprotected contact with case 2 only. An additional 15 staff did not report significant unprotected contact, but because they shared an enclosed office space with case 1 (dotted line), they were deemed to be at higher risk of exposure and were also placed under quarantine.

Given the detection of a COVID-19 cluster with probable HCW–HCW transmission within the hospital, containment measures were instituted. For case 1, initial epidemiology investigations revealed that a large number of MSWs had been potentially exposed, given that the index case had returned to work after medical leave ended and worked for 5 days prior to returning to the staff clinic and diagnosis, while still having mild symptoms. Hence, the decision was made to place all MSWs in our institution on home isolation (a total of 103 HCWs) for an initial period of 24 hours while awaiting further investigation. Because this occurred on a weekend, the disruption to patient care was initially minimal. Subsequently, activity mapping and exhaustive phone interviews were conducted with all affected HCWs to identify their potential exposure and to determine whether any of them were potentially symptomatic over the preceding 2 weeks. In total, 16 of 103 MSWs (15.5%) reported that they had had ARI symptoms over the preceding 2 weeks. However, only 1 of the MSWs had previously reported to the staff clinic before a case of COVID-19 was confirmed, and an alternative diagnosis of dengue had been made, based on serologic testing. As a result, heat maps did not pick up the presence of a cluster in the medical social services department (Fig. 1). Of the 16 symptomatic HCWs, 11 (68.8%) had had significant unprotected contact with case 1 and were thus at risk. Of the HCWs working in the same and adjacent cubicle as the index case, 4 of 8 (50%) had ARI symptoms. All symptomatic HCWs were recalled to the staff clinic or ED for SARS-CoV-2 testing. Of the 16 symptomatic HCWs evaluated, 1 HCW (case 2) tested positive for SARS-CoV-2; the rest were negative. Case 2 worked in the same cubicle as case 1 and sat immediately adjacent, <1 m apart (Fig. 2). They did not socialize outside the work setting. Her onset of symptoms occurred 4 days after case 1 returned to work. Cases 1 and 2 had ~30 minutes of face-to-face conversation per day in the office. Surgical masks were not used in the office but were strictly used for HCW–patient contact. Upon confirmation of case 2, the MSWs were reinterviewed to establish their contact history with case 2; however, because case 2 had presented to the staff clinic at the time of symptom onset, additional contacts were minimal. In total, 49 staff were placed on quarantine (home isolation) based on significant unprotected contact with the 2 cases, constituting 49 of 103 total staff (47.5%) of the medical social services department. No additional cases of COVID-19 were detected among the MSWs in 28 days of follow-up of all exposed staff.

## Discussion

The major finding of this study was that containment of SARS-CoV-2 among HCWs is feasible if vigilance is maintained through ongoing surveillance, and mitigating measures are triggered early upon the detection of a potential cluster. During an ongoing outbreak of SARS-CoV-2, our institution successfully detected and contained a cluster of COVID-19 cases among HCWs, preventing transmission to other HCWs and from HCWs to patients. Early containment of the cluster also minimized the disruption to service. Although almost half of the medical social services department required isolation, our institution did not have to shut down its medical social services department for 2 weeks. We achieved this through an integrated strategy focused on surveillance for early case detection, outbreak investigation and containment, and encouraging compliance with measures for staff protection at the individual level.

Individual-level compliance among HCWs is crucial as the first line of defense during an the SARS-CoV-2 outbreak. Symptom monitoring, early evaluation at the staff clinic, and staying home if unwell were all crucial parts of our institution’s strategy. Indeed, given that persons with COVID-19 can remain contagious even when minimally symptomatic, the risk that HCWs with mild and nonspecific symptoms can serve as a vector of intrahospital disease spread is real, and even 1 infected HCW returning to work has the potential to cause a nosocomial outbreak with devastating consequences.^16^ At our institution, all HCWs with COVID-19 presented with relatively mild upper respiratory tract infection; none presented with pneumonia. Monitoring fever alone is unlikely to be sufficient given that most COVID-19 cases were afebrile, unlike SARS cases, for which temperature monitoring was key.^17^ In our investigation of the MSW cluster, compliance to our institution’s policy of mandatory 5-day medical leave was likely crucial in reducing the potential number of exposed individuals to case 1 when she was most infectious. Emerging data suggest that the viral load in respiratory samples from COVID-19 patients peaks within the first few days after symptom onset before declining.^18^

Addressing presenteeism is critical to ensuring the functionality of staff surveillance in detecting a cluster of COVID-19 among HCWs at an early stage,^2^ and mitigating transmission. Upon subsequent investigation of the MSW cluster, a large number of HCWs reported mild ARI symptoms but had not sought medical evaluation; hence syndromic surveillance was unable to detect the cluster in real time. During an ongoing outbreak, HCWs may experience anxiety when reporting absence from work due to illness^2^ or perceive an obligation to work,^19^ resulting in the phenomenon of “presenteeism,” or work attendance despite illness.^20,21^ Mildness of symptoms is a key factor associated with presenteeism;^22^ this is especially relevant with COVID-19, given mild infection in most individuals.^13^ Institutional policies should encourage work from home (eg, enhancing teleconferencing and telemedicine capabilities) and should not disincentivize individuals from going on medical leave. In our institution, during the outbreak, medical leave for staff with respiratory syndromes was recorded as hospitalization leave, which has a higher annual entitlement than outpatient sick leave.

During a 16-week period of staff surveillance, we detected 14 cases of COVID-19 among HCWs on our campus. Reflecting ongoing community transmission during the prevailing period,^14^ most cases acquired infection from the community or from travel; we detected HCW–HCW spread in only 1 case. The COVID-19 situation was unlike our healthcare system’s previous experience with SARS, in which spread was largely nosocomial.^17^ Although the use of PPE may have helped to prevent patient–HCW transmission, staff surveillance must be vigilant for intrahospital spread occurring outside the usual ward setting, such as office areas and rest areas where HCWs can mingle. Contact among HCWs was shown to be largely assortative in nature; a local study showed that HCWs reported contact with other HCWs of the same type as the next most frequent class of contact after patients.^23^ Thus, initial infections may be concentrated among HCWs of the same type after its introduction into a given setting by an infectious HCW, as occurred in our first staff cluster among MSWs. Given that all HCWs are potentially at risk during a period of ongoing community transmission, surveillance needs to be extended to all staff, not just HCWs who are predominantly ward based (eg, doctors and nurses). This surveillance is especially crucial, given that the potential of transmission from HCWs to patients by allied health professionals should not be underestimated. In local studies, allied health professionals had both the longest cumulative contact time and longest contact episode duration with patients.^23^ All HCW strategies need to include nonclinical and ancillary staff to ensure adequate business continuity for hospitals during a pandemic.^24^ Our detection of HCW clusters in which transmission likely occurred outside work highlights the importance of practicing social distancing outside the work setting.

Our study has several limitations. In our hospital, PCR testing for SARS-CoV-2 was utilized as a diagnostic modality for COVID-19. However, given that the diagnostic yield of PCR testing for SARS-CoV-2 would likely be dependent on the quality and type of respiratory tract sample, similar to results of testing for other coronaviruses,^25^ COVID-19 cases may have been missed due to sampling issues. The follow-up of potentially exposed HCWs to test for seroconversion and further epidemiological studies using serological tests, when such tests are developed, would be useful. Our results also reflect the experience of a healthcare institution in a SARS-CoV-2 outbreak during which the prevailing national strategy was one of containment.^8^ Exhaustive testing, surveillance, and isolation of potentially exposed HCWs may not be feasible in a healthcare system that is overwhelmed.^16^ However, staff surveillance is a crucial component of outbreak control during the containment phase to reduce the likelihood of nosocomial transmission and to sustain confidence and morale in the healthcare system.^2,19,24^

In conclusion, during an ongoing outbreak of COVID-19, our institution successfully detected and contained a cluster of COVID-19 infection among HCWs. This was achieved through an integrated strategy focused on surveillance of ARI symptoms for early case detection, outbreak management, and encouraging compliance at the individual level. Given that most patients with COVID-19 have mild symptoms, addressing presenteeism is crucial to minimizing the number of potentially exposed staff and patients. With ongoing local transmission, staff surveillance must focus on intrahospital spread in nonclinical areas where social mingling of HCWs can occur.
